# Case Report: Melanocyte-associated mTORC1-related molecular features in an atypical large congenital melanocytic naevus

**DOI:** 10.3389/fmed.2026.1962371

**Published:** 2026-10-02

**Authors:** Ruize Tang, Jing Yang, Xingran Liu, Ru-Lin Huang

**Affiliations:** 1 Department of Plastic and Reconstructive Surgery, Shanghai Ninth People’s Hospital, Shanghai Jiao Tong University School of Medicine, Shanghai, China; 2 Shanghai Institute for Plastic and Reconstructive Surgery, Shanghai, China

**Keywords:** congenital melanocytic naevus, molecular profiling, mTORC1 signalling, p-S6, single-cell RNA sequencing, SOX10

## Abstract

Congenital melanocytic naevi (CMN) are usually associated with activating alterations in *NRAS* or *BRAF*, but some extensive lesions lack detectable canonical hotspot variants. We report a 9-year-old boy with a large craniofacial CMN involving the face, scalp, upper eyelid and bulbar conjunctiva, with deep extension, mechanical ptosis and secondary amblyopia. Histology showed benign dermal melanocytic proliferation, and magnetic resonance imaging showed no neurocutaneous melanosis. High-depth whole-exome sequencing detected no canonical *NRAS* or *BRAF* hotspot variant at or above the prespecified reporting threshold. A maternally inherited *TSC2* c.1307C>T (p.Pro436Leu) variant was classified as a variant of uncertain significance. Multiplex immunofluorescence showed greater *p*-S6 positivity among SOX10-positive cells in lesional tissue than in marginal normal skin. Single-cell RNA sequencing showed cell-type-specific variation in mechanistic target of rapamycin complex 1 (mTORC1) scores within the lesion; naevus cells had higher scores than fibroblasts and pericytes but not endothelial or hair follicle cells. These single-patient findings provide descriptive evidence of lesion-associated mTORC1-related molecular features and support pathway-level profiling in atypical CMN, without establishing disease-specific mTORC1 hyperactivation or causality for the *TSC2* variant.

## Introduction

Congenital melanocytic naevi (CMN) are developmental lesions caused by abnormal proliferation and migration of neural crest-derived melanocytes (1). Most large CMN harbour activating alterations in *NRAS* or *BRAF* (2, 3), but canonical hotspot variants are not detected in all cases. Broader molecular profiling may help characterize clinically atypical lesions.

Mechanistic target of rapamycin complex 1 (mTORC1) regulates cell growth, proliferation and metabolism (4, 5). Tuberin, encoded by *TSC2*, negatively regulates mTORC1 signalling, and disruption of the TSC complex can promote tissue overgrowth (4–6). We describe an atypical large craniofacial CMN without a detectable canonical *NRAS* or *BRAF* hotspot variant, in which multimodal profiling identified melanocyte-associated mTORC1-related features. These are presented as descriptive pathway-level findings, not evidence of a new genetic cause of CMN.

## Case description

The clinical and investigative course is summarized in Table 1. The lesion was present at birth, enlarged approximately in proportion to the patient's growth and gradually thickened, most prominently over the forehead and scalp. He had received no previous treatment. There was no family history of CMN, melanoma or tuberous sclerosis complex, and growth and development were otherwise unaffected. His family described him as socially reserved, partly because of concerns about his appearance.

**Table 1:** Timeline of clinical and investigative events.

Time point	Relevant clinical and investigative events
Birth	A congenital pigmented lesion involving the right craniofacial region was present.
Childhood	The lesion enlarged approximately in proportion to growth and gradually thickened, particularly over the forehead and scalp.
Age 9 years	Evaluation documented extensive facial, scalp, eyelid and conjunctival involvement, mechanical ptosis and secondary amblyopia. Magnetic resonance imaging showed no neurocutaneous melanosis or intracranial involvement.
First surgical stage	A tissue expander was implanted for preparation of a prefabricated expanded flap.
Second surgical stage	Most cutaneous naevus tissue was excised and the defect was reconstructed using transfer of the prefabricated expanded flap. Deep extension into the orbicularis oculi and frontalis muscles was observed.
Third surgical stage	Flap revision was performed to improve contour and appearance.
Molecular assessment	High-depth WES detected no canonical *NRAS* or *BRAF* hotspot variant at or above the prespecified reporting threshold. A maternally inherited *TSC2* p.Pro436Leu VUS was identified. p-S6/SOX10 immunofluorescence and scRNA-seq showed lesion-associated mTORC1-related molecular features.
Latest follow-up	More than 1 year after the latest operation, wound healing remained uneventful, with no recurrence in the excised areas or adverse events. Residual naevus remained at the eyelid margin and conjunctiva.

A 9-year-old boy presented with a large, darkly pigmented, rugose plaque involving the right face and scalp, with a projected adult size of 30–40 cm. It was classified as L2, C1, R2, N1, H1 and S1 (7). The lesion extended to the upper eyelid and bulbar conjunctiva and was associated with dermal thickening, mechanical ptosis and secondary amblyopia. The occipital component showed cerebriform thickening and alopecia, and surgery revealed extension into the orbicularis oculi and frontalis muscles (Figure 1A; Supplementary Table S1).

**Figure 1:**
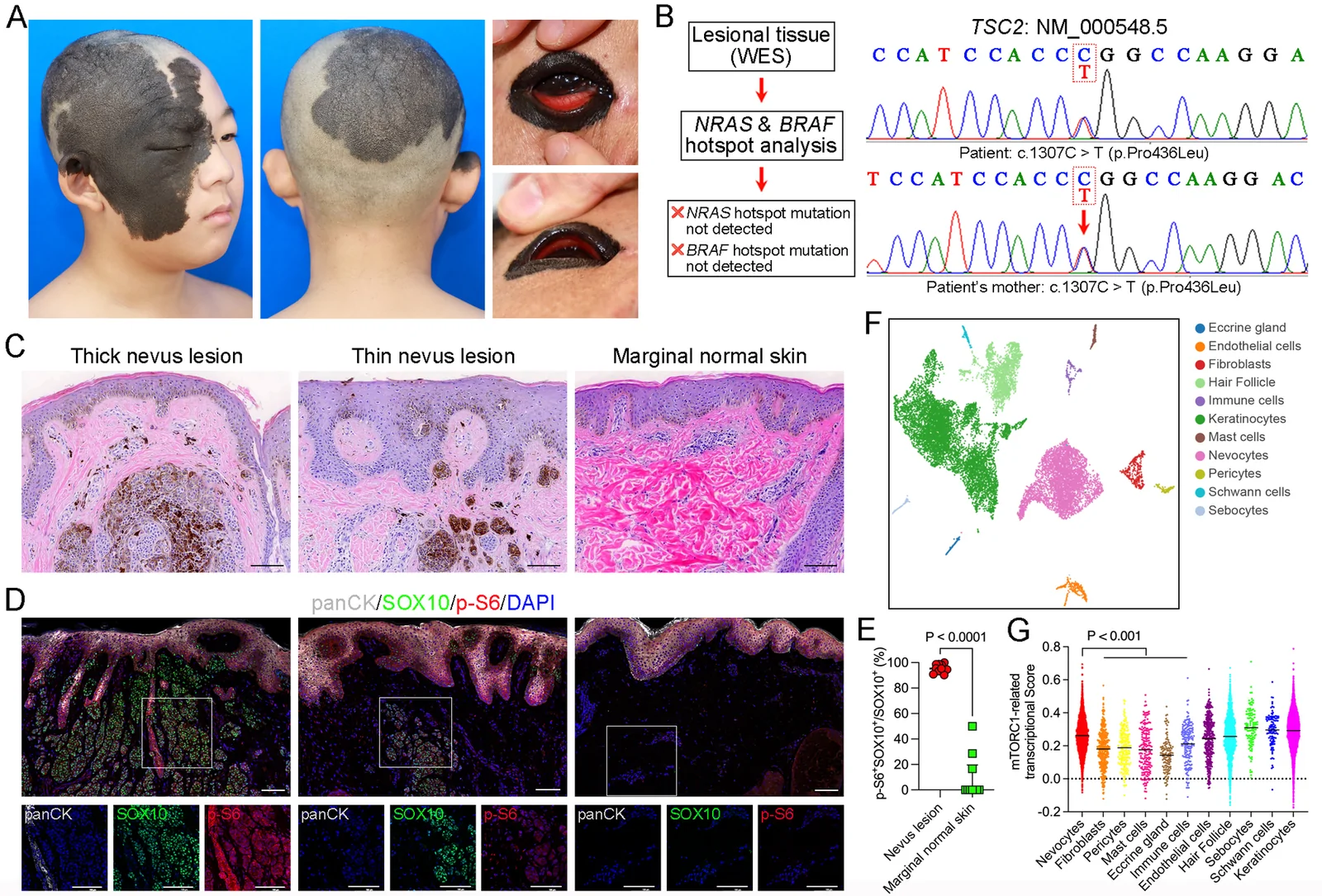
Clinical, histopathological, genomic, immunofluorescence and transcriptomic findings. **(A)** Clinical presentation at 9 years of age. **(B)** High-depth WES detected no canonical *NRAS* or *BRAF* hotspot variant at or above the prespecified reporting threshold; Sanger sequencing confirmed the maternally inherited *TSC2* c.1307C>T (p.Pro436Leu) variant. **(C)** Haematoxylin and eosin staining of thick naevus, thin naevus and marginal normal-skin regions. **(D)** Representative multiplex immunofluorescence for panCK, SOX10, p-S6 and DAPI. **(E)** The p-S6-positive/SOX10-positive proportion was higher in lesional tissue than in marginal normal skin (*P* < 0.0001). **(F)** UMAP of 11 annotated cell populations. **(G)** mTORC1 scores differed across populations (overall Kruskal–Wallis *P* < 0.001). Naevus-cell scores were higher than those of fibroblasts and pericytes (both adjusted *P* < 0.0001) but did not differ from endothelial or hair follicle cells; all comparisons are reported in Supplementary Table S4. Cells and fields were subsamples from one patient, not biological replicates.

Histopathological examination demonstrated dermal melanocytic proliferation without malignant features. Contrast-enhanced magnetic resonance imaging showed no neurocutaneous melanosis or intracranial involvement. Neither the patient nor his mother had clinically apparent features of tuberous sclerosis complex, including facial angiofibromas, hypomelanotic macules, shagreen patches, ungual fibromas or dental enamel pits.

## Diagnostic assessment

High-depth whole-exome sequencing (WES) of lesional tissue and peripheral blood, with Sanger validation and familial testing, identified a maternally inherited heterozygous *TSC2* c.1307C>T (p.Pro436Leu) variant (Figure 1B). Predictive evidence and ACMG/AMP criteria (8) were insufficient for pathogenic or likely pathogenic classification, and the variant was treated as a variant of uncertain significance. No canonical *NRAS* or *BRAF* hotspot variant was detected at or above the prespecified reporting threshold. Methods, coverage and locus-level results are provided in Supplementary Method S2 and Supplementary Tables S2, S3.

Multiplex immunofluorescence showed p-S6 staining in SOX10-positive naevus cells (Figure 1D) and a higher p-S6-positive/SOX10-positive proportion in lesional tissue than in marginal normal skin (*P* < 0.0001; Figure 1E). Quantitative details are provided in Supplementary Method S1 and Supplementary Table S5. This intra-specimen comparison does not establish disease-specific mTORC1 hyperactivation.

Single-cell RNA sequencing (scRNA-seq) identified 11 annotated cell populations within the lesion (Figure 1F). In an exploratory cell-level analysis, mTORC1 scores varied across populations; naevus cells had higher scores than fibroblasts and pericytes but did not differ from endothelial or hair follicle cells (Figure 1G). Other comparisons and effect sizes are reported in Supplementary Table S4, with methods in Supplementary Method S3. Because all cells came from one lesion, these findings describe intra-lesional variation rather than patient-level replication.

## Therapeutic intervention

The patient underwent three staged operations: tissue-expander implantation, excision with prefabricated expanded-flap transfer, and flap revision. Staging was required by the lesion's extent and the reconstructive approach. Most cutaneous naevus was excised; residual tissue remained at the eyelid margin and conjunctiva. Further flap revision is planned. The molecular findings did not alter management, and no pathway-directed treatment was administered.

## Follow-up and outcomes

More than 1 year after the latest operation, wounds had healed without recurrence in the excised areas. Mechanical ptosis and eyelid closure had improved, whereas visual acuity, the ocular surface and conjunctival involvement were unchanged. Residual naevus remained at the eyelid margin and conjunctiva. No adverse or unanticipated event occurred during staged treatment or follow-up. Continued clinical surveillance is planned.

## Discussion

Congenital melanocytic naevi are genetically heterogeneous. Most are associated with activating *NRAS* or *BRAF* alterations (2, 3), whereas the present lesion had no detectable canonical hotspot variant at or above the WES reporting threshold. This contrast supports broader molecular characterization in selected atypical cases.

Multiplex immunofluorescence showed greater p-S6 immunoreactivity in lesional SOX10-positive cells than in marginal normal skin. The scRNA-seq analysis showed cell-type-specific variation in mTORC1 scores within the lesion: naevus cells had higher scores than fibroblasts and pericytes but not endothelial cells, while several non-melanocytic populations had equivalent or higher scores. Together, these findings support lesion-associated mTORC1-related features but not uniform, disease-specific pathway hyperactivation.

The inherited *TSC2* variant remains a contextual finding. No additional *TSC2* SNV/InDel or significant copy-number alteration was detected; loss of heterozygosity, allelic imbalance and variant-specific function were not assessed (Supplementary Method S2 and Supplementary Table S2). Its coexistence with the mTORC1-related findings raises a testable hypothesis, but maternal inheritance from an apparently unaffected carrier, the absence of tuberous sclerosis complex features and the lack of functional evidence preclude causal inference.

A strength of this case is the integration of WES, immunofluorescence and scRNA-seq. Contemporary assessment of challenging melanocytic lesions nevertheless relies on integrated clinicopathological evaluation, with molecular and immunohistochemical assays used as ancillary tools (9). Here, these assays refined molecular characterization but did not change the benign pathological diagnosis, establish malignant transformation or guide treatment.

This single-patient report cannot establish whether the observed features recur in atypical CMN. The scRNA-seq analysis lacked independent normal melanocyte and CMN controls, and cells and microscopic fields were subsamples rather than biological replicates. High-depth WES may still miss very-low-level mosaic, non-hotspot or structural variants outside its analytical scope. The relevance of the inherited *TSC2* variant remains unresolved without loss-of-heterozygosity, allelic-imbalance or functional analyses. Larger controlled cohorts and longer follow-up are needed.

In conclusion, this atypical large CMN showed greater p-S6 immunoreactivity in lesional SOX10-positive cells than in marginal normal skin and cell-type-specific mTORC1 scores within the lesion. These descriptive findings support multimodal pathway-level characterization but do not establish disease-specific mTORC1 hyperactivation or causality for the *TSC2* variant.

## Data Availability

The raw individual-level WES and scRNA-seq datasets are not readily available because they contain potentially identifiable genomic and transcriptomic data from a single paediatric participant. Requests to access these datasets should be directed to the corresponding author, Ru-Lin Huang (huangrulin@sjtu.edu.cn), and will be considered subject to institutional and ethical approval. Data supporting the reported analyses are included in the article and Supplementary Material.

## Patient Perspective

The patient and his family were satisfied with the reconstructive outcome and planned to continue flap refinement to further improve appearance.

## References

[1] Kinsler VA O'Hare P Bulstrode N Calonje JE Chong WK Hargrave D. Melanoma in congenital melanocytic naevi. Br J Dermatol. (2017) 176:1131–43. 10.1111/bjd.15301 28078671 PMC5484991

[2] Shain AH Bastian BC. From melanocytes to melanomas. Nat Rev Cancer. (2016) 16:345–58. 10.1038/nrc.2016.37 27125352

[3] Charbel C Fontaine RH Malouf GG Picard A Kadlub N El-Murr N. *NRAS* mutation is the sole recurrent somatic mutation in large congenital melanocytic naevi. J Invest Dermatol. (2014) 134:1067–74. 10.1038/jid.2013.429 24129063

[4] Yang H Yu Z Chen X Li J Li N Cheng J. Structural insights into TSC complex assembly and GAP activity on rheb. Nat Commun. (2021) 12:339. 10.1038/s41467-020-20522-4 33436626 PMC7804450

[5] Saxton RA Sabatini DM. mTOR signaling in growth, metabolism, and disease. Cell. (2017) 168:960–76. 10.1016/j.cell.2017.02.004 28283069 PMC5394987

[6] Henske EP Jóźwiak S Kingswood JC Sampson JR Thiele EA. Tuberous sclerosis complex. Nat Rev Dis Primers. (2016) 2:16035. 10.1038/nrdp.2016.35 27226234

[7] Krengel S Scope A Dusza SW Vonthein R Marghoob AA Myskowski PL. New recommendations for the categorization of cutaneous features of congenital melanocytic nevi. J Am Acad Dermatol. (2013) 68:441–51. 10.1016/j.jaad.2012.05.043 22982004

[8] Richards S Aziz N Bale S Bick D Das S Gastier-Foster J. Standards and guidelines for the interpretation of sequence variants: a joint consensus recommendation of the American college of medical genetics and genomics and the association for molecular pathology. Genet Med. (2015) 17:405–24. 10.1038/gim.2015.30 25741868 PMC4544753

[9] Pepe L Fiorentino V Pizzimenti C Martini M Santarpia M Fazio A. The diagnostic and prognostic role of combined p16 and MTAP immunohistochemistry in melanocytic tumors of uncertain malignant potential: a comprehensive review and clinical practice analysis. Int J Mol Sci. (2026) 27:971. 10.3390/ijms27020971 41596618 PMC12841788

