# Supplementary material for: Case Report: Melanocyte-associated mTORC1-related molecular features in an atypical large congenital melanocytic naevus
Source: Front Med (Lausanne). 2026 Oct 2;13:1962371. doi: 10.3389/fmed.2026.1962371 (PMC13671986; doi:10.3389/fmed.2026.1962371)
Supplement: Supplementary file 1 [file Supplementaryfile1.docx]

# Supplementary Material

## Supplementary Methods

### Method S1. Multiplex immunofluorescence staining and quantitative analysis

Formalin-fixed, paraffin-embedded sections from thick naevus, thin naevus and marginal normal-skin regions were stained in the same run for panCK, SOX10, p-S6 and DAPI. Thick and thin lesional regions were distinguished by the depth and density of dermal SOX10-positive naevus-cell proliferation on matched hematoxylin and eosin and immunofluorescence images; marginal normal skin was adjacent histologically unremarkable skin without dermal naevus nests. The selected tissue regions were confirmed by a pathologist. Representative images from all three regions are shown, whereas quantitative analysis compared the combined naevus lesion with marginal normal skin. Twenty non-overlapping microscopic fields (10 lesional and 10 marginal normal-skin fields) from three non-adjacent sections were acquired using identical settings. SOX10-positive nuclear regions of interest (ROIs) were identified by DAPI-constrained automated segmentation without using the p-S6 channel; each ROI was expanded by 5 pixels for measurement of mean p-S6 fluorescence. A fixed threshold of 1 arbitrary unit, derived from the negative control, was applied uniformly. The percentage of p-S6-positive/SOX10-positive cells among all SOX10-positive cells was calculated for each field. Field-level distributions were summarized as median and interquartile range (IQR) and compared using an exact two-sided Mann-Whitney test in GraphPad Prism 10. The fields were spatial subsamples from one patient rather than independent biological replicates.

### Method S2. Whole-exome sequencing and variant analysis

Raw reads were processed using fastp (v0.23.2) and aligned using BWA-MEM2 (v2.2.1). Lesional tissue and peripheral-blood samples were analysed separately using GATK HaplotypeCaller (v4.2.3.0), and the resulting SNV/InDel calls were subsequently compared. Quality-control reports, variant call format (VCF) files and binary alignment map (BAM) files were used for sequencing-quality and variant assessment. Mean on-target depths were 225.18× for lesional tissue and 182.25× for peripheral blood. In lesional tissue, 99.91% and 99.80% of targeted bases were covered at ≥10× and ≥20×, respectively; 99.74% of targeted bases in peripheral blood were covered at ≥20×. Candidate variants were retained at a total depth of ≥5×, with ≥3 alternate-supporting reads and a variant allele fraction of ≥10%. Variants were annotated using Variant Effect Predictor (VEP) v112, and variants with a population allele frequency of ≥1% in gnomAD v4.1 were excluded. Canonical *NRAS* and *BRAF* hotspot positions were evaluated within the WES analysis, and no canonical hotspot variant was detected at or above the prespecified reporting threshold. Locus-level depth, reference-read count, alternate-read count, and VAF data for the evaluated hotspots are provided in **Supplementary Table S3**. Canonical *NRAS* and *BRAF* hotspot positions and candidate variants were manually reviewed using IGV (v2.19.8). A formally validated analytical limit of detection for low-level mosaic variants was not established for this research WES workflow. Given the high mean sequencing depth, the analysis provides reasonable confidence that a canonical hotspot variant was not present at a variant allele fraction at or above the prespecified bioinformatic reporting threshold; however, very-low-level mosaic variants below locus-specific coverage or reporting thresholds cannot be entirely excluded. The *TSC2* c.1307C>T (p.Pro436Leu) variant showed variant allele fractions of 51.12% in lesional tissue and 48.94% in peripheral blood, was confirmed by Sanger sequencing and was shown to be maternally inherited. It was classified as a VUS under ACMG/AMP criteria.

### Method S3. Single-cell RNA sequencing and analysis

Single-cell RNA sequencing was performed on microdissected lesional tissue. A total of 23,271 cells were initially identified. Cells with ≤500 or ≥6,000 detected genes, ≥30,000 unique molecular identifier (UMI) counts, or ≥15% mitochondrial transcripts were excluded. Potential doublets were removed using Scrublet v0.2.3 (doublet-score cutoff, 0.25), leaving 16,107 cells. Total-count normalization to 10,000 counts per cell and log1p transformation preceded variable-feature selection, principal-component analysis, graph-based clustering and UMAP visualization in Scanpy v1.12 under Python 3.12.7. Populations were annotated using established lineage markers (**Supplementary Table S4**). No between-sample integration or batch correction was performed because one lesion was analysed. The mTORC1 score was calculated from the 24-gene REACTOME_MTORC1_MEDIATED_SIGNALLING gene set from the Molecular Signatures Database (MSigDB), C2 Reactome collection, version 2026.1.Hs, using scanpy.tl.score_genes on the normalized matrix (control-gene set size, 50; 25 expression bins; random seed, 42). This score reflects relative transcriptional enrichment and is not a direct biochemical measurement of mTORC1 activity. Population distributions were compared using the Kruskal-Wallis test followed by Dunn's test in GraphPad Prism 10. Cliff's δ was calculated from cell-level distributions with naevus cells as the reference; 95% percentile confidence intervals used 5,000 stratified bootstrap resamples. These were exploratory within-lesion analyses, not patient-level biological replication.

## Supplementary Table 1

**Table S1. Clinical context, molecular findings, and evidentiary interpretation of the index case.**

| **Domain** | **Variable** | **Finding** | **Evidentiary interpretation** |
| --- | --- | --- | --- |
| Clinical context | Patient | 9-year-old boy with a large congenital melanocytic naevus involving the right face and scalp | Index case with an extensive and clinically atypical craniofacial lesion |
| Clinical context | Projected adult size | 30–40 cm | Consistent with a large congenital melanocytic naevus |
| Clinical context | Consensus classification | L2, C1, R2, N1, H1 and S1 | Documents clinical extent and associated surface features |
| Clinical context | Periocular involvement | Upper eyelid and bulbar conjunctiva, with mechanical ptosis and secondary amblyopia | Clinically relevant functional involvement |
| Clinical context | Occipital component | Cerebriform thickening with alopecia | Unusual clinical architecture |
| Clinical context | Deep tissue involvement | Extension into the orbicularis oculi and frontalis muscles during excision | Indicates deep local involvement |
| Clinical context | Magnetic resonance imaging | No neurocutaneous melanosis or intracranial involvement detected | No radiological evidence of central nervous system involvement at evaluation |
| Clinical context | Histopathology | Dermal melanocytic proliferation without malignant features | Consistent with CMN; no malignancy identified |
| Canonical CMN alterations | *NRAS* hotspot status | No canonical *NRAS* hotspot variant detected at or above the prespecified reporting threshold by high-depth WES | No canonical *NRAS* hotspot alteration was detected within the assay’s reporting scope. |
| Canonical CMN alterations | *BRAF* hotspot status | No canonical *BRAF* hotspot variant detected at or above the prespecified reporting threshold by high-depth WES | No canonical *BRAF* hotspot alteration was detected within the assay’s reporting scope. |
| *TSC2* finding | Gene and transcript | *TSC2*, NM_000548.5 | Encodes tuberin, a negative regulator of mTORC1 signalling |
| *TSC2* finding | Variant | c.1307C>T; p.Pro436Leu | Heterozygous missense variant |
| *TSC2* finding | Specimens detected | Peripheral blood and lesional tissue | Consistent with a germline variant |
| *TSC2* finding | Inheritance | Maternally inherited | An apparently unaffected carrier weakens a simple genotype–phenotype interpretation |
| *TSC2* finding | gnomAD v4.1.0 | Absent | Supports population rarity under ACMG PM2; rarity alone does not establish pathogenicity |
| *TSC2* finding | CADD score | 25.5 | Computational prediction only; does not demonstrate functional impairment |
| *TSC2* finding | AlphaMissense score | 0.856 | Computational prediction only; contributes to PP3 evidence |
| *TSC2* finding | EVE score | 0.721 | Computational prediction only; contributes to PP3 evidence |
| *TSC2* finding | ACMG evidence | PM2 and PP3 | Insufficient for classification as pathogenic or likely pathogenic |
| *TSC2* finding | Variant classification | Variant of uncertain significance | Contextual genetic finding, not a proven cause or modifier of the naevus |
| TSC clinical assessment | Patient | No clinically apparent facial angiofibromas, hypomelanotic macules, shagreen patches, ungual fibromas or dental enamel pits | No clinically apparent tuberous sclerosis complex |
| TSC clinical assessment | Mother | No clinically apparent features of tuberous sclerosis complex | Does not support cosegregation with a syndromic phenotype |
| Pathway-level finding | p-S6/SOX10 immunofluorescence | Median p-S6-positive proportion among SOX10-positive cells: 95.38% in lesion versus 0.00% in marginal normal skin; exact Mann-Whitney U=0, P<0.0001 | Exploratory intra-specimen field-level difference; not proof of disease-specific mTORC1 hyperactivation |
| Pathway-level finding | Single-cell RNA sequencing | 16,107 cells retained after quality control and classified into 11 cell populations | Enabled exploratory cell-type-resolved pathway-score analysis within the index lesion |
| Pathway-level finding | mTORC1-related transcriptional score | mTORC1 scores differed across cell populations; naevus cells had higher scores than fibroblasts and pericytes but not endothelial or hair follicle cells | Describes cell-type-specific differences within one lesion; does not establish disease-specific hyperactivation |
| Pathway-level finding | Statistical analysis | Kruskal-Wallis H=974.20, P<0.0001, followed by Dunn's multiple-comparisons test | Cells originated from one lesion and were not independent patient-level biological replicates; pairwise results are reported in **Supplementary Table S4** |
| Interpretive limitation | Causal genotype–phenotype relationship | Not established | Available data do not demonstrate that the *TSC2* variant caused the CMN |
| Interpretive limitation | Somatic second hit | No additional *TSC2* SNV/InDel identified | A lesion-specific second genetic event cannot be confirmed or excluded |
| Interpretive limitation | Copy-number variation or loss of heterozygosity | No significant *TSC2* regional CNV alteration was detected; LOH and allelic-imbalance analyses were not performed. | A *TSC2*-related two-hit mechanism could neither be confirmed nor excluded. |
| Interpretive limitation | Functional validation of p.Pro436Leu | Not performed | Direct impairment of tuberin function was not demonstrated |
| Interpretive limitation | Independent scRNA-seq control | Normal melanocytes and an independent CMN control were not included | The transcriptional-score comparison was restricted to cell populations within one lesion |
| Interpretive limitation | Replication in additional cases | Not performed | Findings are hypothesis-generating and cannot define a recurrent molecular subgroup |
| Clinical management | Staged reconstruction | Three operations: tissue-expander implantation, prefabricated expanded-flap transfer and flap revision | Most cutaneous naevus was excised; residual eyelid-margin and conjunctival naevus remains |
| Clinical outcome | Latest follow-up | >1 year after the most recent operation; uneventful healing, no recurrence in excised areas, improved ptosis and eyelid closure | Visual acuity, ocular surface and conjunctival involvement were unchanged |
| Clinical outcome | Adverse events | No adverse or unanticipated event to date | No documented perioperative or new ophthalmological complication |

**Abbreviations:** ACMG, American College of Medical Genetics and Genomics; *BRAF*, B-Raf proto-oncogene; CADD, Combined Annotation Dependent Depletion; CMN, congenital melanocytic naevus; EVE, evolutionary model of variant effect; mTORC1, mechanistic target of rapamycin complex 1; *NRAS*, neuroblastoma RAS viral oncogene homolog; p-S6, phosphorylated ribosomal protein S6; scRNA-seq, single-cell RNA sequencing; SOX10, SRY-box transcription factor 10; TSC, tuberous sclerosis complex; *TSC2*, tuberous sclerosis complex 2; UMI, unique molecular identifier; VEP, Variant Effect Predictor; VUS, variant of uncertain significance; WES, whole-exome sequencing.

## Supplementary Table 2

**Table S2.** **WES quality-control and locus-level reporting.**

| **Specimen** | **Variant/locus** | **Mean on-target depth** | **Locus coverage** | **Alternate-supporting reads** | **Observed VAF** | **Reporting threshold** | **Result/interpretation** |
| --- | --- | --- | --- | --- | --- | --- | --- |
| Lesional tissue | Canonical *NRAS* hotspots | 225.18× | See **Table S3** | See **Table S3** | See **Table S3** | VAF ≥10%; depth ≥5×; alternate reads ≥3 | No variant detected at or above the reporting threshold |
| Lesional tissue | Canonical *BRAF* hotspots | 225.18× | See **Table S3** | See **Table S3** | See **Table S3** | VAF ≥10%; depth ≥5×; alternate reads ≥3 | No variant detected at or above the reporting threshold |
| Lesional tissue | *TSC2* NM_000548.5:c.1307C>T (p.Pro436Leu) | 225.18× | 268× | 137 | 51.12% | VAF ≥10%; depth ≥5×; alternate reads ≥3 | Heterozygous VUS; confirmed by Sanger sequencing |
| Peripheral blood | *TSC2* NM_000548.5:c.1307C>T (p.Pro436Leu) | 182.25× | 331× | 162 | 48.94% | VAF ≥10%; depth ≥5×; alternate reads ≥3 | Heterozygous; consistent with germline origin |

Overall, 99.91% and 99.80% of lesional targeted bases were covered at ≥10× and ≥20×, respectively, and 99.74% of peripheral-blood targeted bases were covered at ≥20×. Very-low-level mosaic, non-hotspot and structural variants outside the reliable analytical scope cannot be entirely excluded.

**Abbreviations**: BAM, binary alignment map; VAF, variant allele fraction; VCF, variant call format; VUS, variant of uncertain significance; WES, whole-exome sequencing.

## Supplementary Table 3

**Table S3.** **Sequencing data for canonical *NRAS* and *BRAF* hotspot positions in lesional tissue and peripheral blood**.

| **Gene** | **Hotspot** | **GRCh38 position** | **Lesional tissue** | **Peripheral blood** |
| --- | --- | --- | --- | --- |
| *BRAF* | V600 | chr7:140753335 | DP 421; REF C=421; ALT=0; VAF 0% | DP 390; REF C=390; ALT=0; VAF 0% |
| *BRAF* | V600 | chr7:140753336 | DP 417; REF A=417; ALT=0; VAF 0% | DP 392; REF A=392; ALT=0; VAF 0% |
| *BRAF* | V600 | chr7:140753337 | DP 415; REF C=415; ALT=0; VAF 0% | DP 391; REF C=391; ALT=0; VAF 0% |
| *NRAS* | Q61 | chr1:114713907 | DP 278; REF T=278; ALT=0; VAF 0% | DP 291; REF T=291; ALT=0; VAF 0% |
| *NRAS* | Q61 | chr1:114713908 | DP 279; REF T=279; ALT=0; VAF 0% | DP 289; REF T=289; ALT=0; VAF 0% |
| *NRAS* | Q61 | chr1:114713909 | DP 276; REF T=276; ALT=0; VAF 0% | DP 288; REF T=288; ALT=0; VAF 0% |
| *NRAS* | G13 | chr1:114716122 | DP 341; REF A=340; ALT C=1; VAF 0.29% | DP 366; REF A=366; ALT=0; VAF 0% |
| *NRAS* | G13 | chr1:114716123 | DP 348; REF C=348; ALT=0; VAF 0% | DP 375; REF C=375; ALT=0; VAF 0% |
| *NRAS* | G13 | chr1:114716124 | DP 348; REF C=348; ALT=0; VAF 0% | DP 379; REF C=379; ALT=0; VAF 0% |
| *NRAS* | G12 | chr1:114716125 | DP 347; REF A=347; ALT=0; VAF 0% | DP 379; REF A=379; ALT=0; VAF 0% |
| *NRAS* | G12 | chr1:114716126 | DP 351; REF C=351; ALT=0; VAF 0% | DP 379; REF C=378; ALT G=1; VAF 0.26% |
| *NRAS* | G12 | chr1:114716127 | DP 353; REF C=353; ALT=0; VAF 0% | DP 377; REF C=377; ALT=0; VAF 0% |

## Supplementary Table 4

**Table S4.** **Cell numbers, representative lineage-associated markers and exploratory mTORC1-related transcriptional-score comparisons.**

| Cell population | Cells, n | Median score (Q1-Q3) | Representative annotation markers | Mean rank | Dunn comparison with naevus cells | Cliff's δ (95% CI) |
| --- | --- | --- | --- | --- | --- | --- |
| Naevus cells | 4,778 | 0.2594 (0.1920-0.3318) | *MLANA*, *PMEL*, *TYR*, *SOX10* | 7547.011 | Reference | Reference |
| Eccrine gland cells | 124 | 0.1412 (0.0733-0.1931) | *KRT8*, *KRT18*, *KRT19*, *AQP5* | 3123.363 | Naevus cells higher; adjusted P<0.0001 | 0.605 (0.516-0.687) |
| Endothelial cells | 321 | 0.2479 (0.1513-0.3406) | *PECAM1*, *VWF*, *EMCN* | 7053.234 | Not significant; adjusted P=0.6551 | 0.076 (0.005-0.150) |
| Fibroblasts | 379 | 0.1833 (0.1000-0.2587) | *COL1A1*, *COL1A2*, *DCN*, *LUM* | 4522.507 | Naevus cells higher; adjusted P<0.0001 | 0.408 (0.346-0.465) |
| Hair follicle cells | 1,876 | 0.2593 (0.1825-0.3412) | *KRT15*, *KRT17*, *SOX9* | 7479.242 | Not significant; adjusted P>0.9999 | 0.015 (-0.016 to 0.047) |
| Immune cells | 173 | 0.2266 (0.1334-0.2947) | *PTPRC*, *CD3D*, *LST1* | 5804.023 | Naevus cells higher; adjusted P<0.0001 | 0.229 (0.146-0.314) |
| Keratinocytes | 7,885 | 0.2975 (0.2239-0.3681) | *KRT5*, *KRT14*, *KRT1*, *KRT10* | 8938.780 | Naevus cells lower; adjusted P<0.0001 | -0.179 (-0.198 to -0.158) |
| Mast cells | 174 | 0.1679 (0.0712-0.2808) | *TPSAB1*, *KIT*, *CPA3* | 4739.897 | Naevus cells higher; adjusted P<0.0001 | 0.384 (0.297-0.479) |
| Pericytes | 168 | 0.1927 (0.1033-0.2669) | *RGS5*, *CSPG4*, *PDGFRB* | 4806.946 | Naevus cells higher; adjusted P<0.0001 | 0.371 (0.286-0.450) |
| Schwann cells | 114 | 0.3022 (0.2365-0.3680) | *S100B*, *SOX10*, *PLP1*, *MPZ* | 9172.307 | Naevus cells lower; adjusted P=0.0023 | -0.216 (-0.314 to -0.123) |
| Sebocytes | 115 | 0.3123 (0.2461-0.3891) | *PPARG*, *PLIN2*, *MGST1* | 9611.809 | Naevus cells lower; adjusted P<0.0001 | -0.264 (-0.373 to -0.164) |

The overall Kruskal-Wallis test was significant (H=974.20, P<0.0001), followed by Dunn's multiple-comparisons test. Cliff's δ compares naevus cells with each listed population; positive values indicate higher scores in naevus cells. The 95% confidence intervals are unadjusted percentile bootstrap intervals based on 5,000 cell-level resamples within each population and do not necessarily correspond to Dunn-adjusted significance. All cells came from one lesion and were not biological replicates. Scores were calculated as described in **Supplementary Method S3**.

## Supplementary Table 5

**Table S5.** **Quantitative p-S6/SOX10 immunofluorescence analysis.**

| **Tissue region** | **Fields, n** | **SOX10^+^ cells, n** | **p-S6^+^SOX10^+^ cells, n** | **Field-level positive proportion, median (IQR), %** |
| --- | --- | --- | --- | --- |
| Naevus lesion | 10 | 4,330 | 4,148 | 95.38 (92.73-98.69) |
| Marginal normal skin | 10 | 53 | 5 | 0.00 (0.00-19.64) |

Twenty non-overlapping fields from three non-adjacent sections were analysed. The field-level distributions were compared using an exact two-sided Mann-Whitney test (U=0, P<0.0001). Fields were spatial subsamples from one patient rather than independent biological replicates.

**Abbreviation**: IQR, interquartile range.
